# Determination of carcinogenic benzo(a)pyrene in heat treated black tea samples from Turkey by using HPLC-fluorescence detection system

**DOI:** 10.1007/s13197-024-06031-y

**Published:** 2024-07-20

**Authors:** Seker Fatma Aygun, Cigdem Dikbas, Zebron Nchimunya Tembo

**Affiliations:** 1 Science and Arts Faculty, Department of Chemistry, Ondokuz Mayis University, Samsun, Turkey; 2 School of Natural and Applied Sciences, Department of Chemistry, Mulungushi University, Kabwe, Zambia

**Keywords:** Black tea, BaP, HPLC, Microwave assisted extraction

## Abstract

A rapid, simple, and cost-efficient extraction method was developed for evaluating and screening benzo(a)pyrene (BaP) in tea samples by using high performance liquid chromatography (HPLC) with coupled fluorescence detector (FLD) in order to obtain the best extraction performance. In this study, it was observed that when optimized using microwave assisted extraction (MAE) method was performed twice for 2 min using 10 mL n-hexane: acetonitrile (1:3, v/v). The recoveries for BaP in tea were found to be 97 ± 2; 83 ± 8 and 92 ± 6%, respectively. Using classical solid-liquid extraction (CSLE), it was observed that when the extraction procedure was performed twice for 4 min with 10 mL n-hexane: acetonitrile (3:1,v/v). The recoveries for BaP in tea were found to be 104 ± 5; 81 ± 9 and 86 ± 8%, respectively. The levels of BaP in tea were found to change in the range from 0.75 to 0.83 µg kg^-1^ (MAE) and 0.72 to 0.86 µg kg^-1^ (CSLE). Although the matrix of tea is complex, BaP was detectable without doing any cleaning up procedures.

## Introduction

Tea is one of the most consumed beverages in many cultures of the world. The practice of daily consumption of tea requires constant production the production of the commodity producing countries. Turkey is the sixth largest producer of tea in the world after China, India, Sri Lanka, Kenya and Indonesia. More than 140,000 tons of black tea are consumed in Turkey every year (Aksuner et al. 2012). Tea is a kind of beverage obtained by boiling the treated plant leaves. It is the most consumed after water in the world and it ranks first place among caffeine-containing drinks (Khiadani et al. 2013). The studies conducted all over the world as well as in Turkey are focussed on the positive effects of tea consumption on health such as cholesterol reduction, antioxidant effect, protection against cancer and prevention of heart diseases (Elmastasa et al. 2018). Therefore, it is important to examine not only the positive effects but also negative effects (Girelli et al. 2017). Detrimental effects may arise due to intake of contaminants such as some heavy metals, pesticides and polyaromatic hydrocarbons (PAHs) which can be absorbed in the leaves of the tea plant via air, water or soil. It is important to note that PAHs can also contaminate teas during processing of the tea leaves (Türkmen 2007).

Polyaromatic hydrocarbons (PAHs) are hydrophobic organic compounds with two or more benzene rings. PAHs are compounds that are toxic and carcinogenic, usually occurred by incomplete combustion of organic compouds. It has been proved by studies that cooking at high temperatures especially in foods causes the formation of PAH (Alver et al. 2012). PAHs can be found in water and everyday foods such as vegetables, fruits, fat, smoked meat, charcoal grilled meat and black tea (Aygun and Bagcevan 2019; Aygun and Kabadayi 2005; Karyab et al. 2013; Nworah et al. 2019). Tea leaves have a large surface area and can absorb PAHs, especially from air. Particularly effective sources of PAHs in black tea are due to the combustion gases resulting from the combustion of wood, coal, oil used for drying tea (Girelli et al. 2017). As an indicator of the presence of other PAHs, Benzo(a)pyrene (BaP) can be said one of the most carcinogenic PAHs. High-performance liquid chromatography (HPLC) is a widely used for the separation and quantitative determination of many different classes of compounds (such as amino acids, proteins, hydrocarbons, carbohydrates, drugs, and compounds that can easily degenerate by temperature). PAHs can be analyzed using HPLC devices with fluorescence or UV absorbance detectors, connected phase columns that are effective in separating PAHs, and systems capable of gradient elution. Fluorescence detectors are especially preferred in the analyzes because of their more sensitivity and selectivity (Aygun 2005).

The aim of the study is to develop a simple and inexpensive extraction technique in order to detect and determine BaP content in black tea samples by HPLC. For this purpose, a number of extraction parameters (choice of solvent type, solvent volume and extraction time) in (CSLE) and MAE were optimized and these two methods were compared.

## Materials and methods

### Reagents

98% purity benzo (a) pyrene (BaP) standard purchased from Sigma Chemical Co. HPLC purity acetonitrile (Merck), dichloromethane (Merck), n-hexane (Merck), acetone (Merck) solutions.

### Instrumentation

BaP was analyzed by the Shimadzu (Japan) brand HPLC coupled with a Shimadzu RF-20 A Spectrofluorometer detector with a reverse-phase C18 column (GL Science inertsil ODS-3 4.6 mm x 250 mm i.d., 5 μm particle size). The flow rate of the mobile phase was kept at 1.0 mL min^− 1^. The temperature of the column oven was maintained at 40 °C.

### Preparation of tea samples by extraction method

Black teas of various brands produced between 2016 and 2017 were obtained from markets in Samsun, Turkey. Approximately one gram of tea samples was placed in the flasks and 10 mL of n-hexane: acetonitrile (3:1, 1:3, v/v respectively) were added to them and the flasks containing the samples were extracted with microwave assisted and classical extraction techniques for 2 min and 4 min. The microwave assisted extraction procedure was carried out using a household microwave oven emitting 180 W. During the extraction using polar solvents, flasks containing 250 mL of water were also placed in the oven to prevent the samples from overheating. The solvents obtained from both extractions were removed by rotary evaporation. The residue obtained after extractions were dissolved in n-hexane: acetonitrile and this extracts were air dried. The last residues obtained were dissolved in 8 mL of acetonitrile. The prepared samples were centrifuged at 2000 rpm to prevent column clogging and then made available for HPLC analysis (Dikbas 2018).

### Preparation of tea brews samples by extraction method

12 g of dry tea was placed in the pot and 360 mL of drinking water was added. The teapot was left to infuse for 15 min on boiling water containing 1 L of water. The brewed liquid tea phase was filtered off from the tea pulp. 120 mL portions of the separated liquid phase were taken up in three flasks and heated in the microwave until the water phases had evaporated. 40 mL of n-hexane: acetonitrile (1:3,v/v) was added to the solids remaining on the bottom of the flasks and extracted twice in the microwave (180 W power for 2 min) and the solvent of the extracts obtained by filtration were removed by rotary evaporator. The most recently obtained residues were dissolved in 8 mL of acetonitrile and the prepared samples were centrifuged to prepare HPLC for analysis (Dikbas 2018).

The procedure above was also applied for classical solid liquid extraction. Only 40 mL of n-hexane-acetonitrile (3:1, v/v) was added as extraction solvent (Dikbas 2018).

### HPLC analysis

Chromatographic separation conditions for the analyzes included a sample injection volume of 2 µL, a column temperature of 40 °C and flow rate of 1 mL min^− 1^. An isocratic mode with a mobile phase of 100% acetonitrile was employed. A fluorescence detector with a signal excitation set at 280 nm and an emission of 400 nm was selected. The detector had a 7 min duration for excitation and emission. Calibration curves were then plotted using standard solutions of BaP prepared at different concentrations. The standard calibrating solutions of BaP of 0.125 to 0.250 µg L^− 1^ concentrations in acetonitrile with three replicates resulting in correlation coefficients ranging from 0.9971 (for CSLE) to 0.9996 (for MAE) were prepared by diluting stock BaP solution.

### Recovery, LOD and LOQ of BaP from tea samples

In order to obtain % recovery values ​​in the analysis of the tea samples, BaP standard solutions were added to the dry tea samples at three different concentrations (159.0; 12.7 and 1.3 µg kg^− 1^) before extraction and analysis of each sample following the application of the selected procedure (extraction, dissolution of the obtained residue in acetonitrile and analysis with HPLC). The limit of detection (LOD) and the limit of quantification (LOQ) were calculated in relation with the signal to noise ratios of 3 and 10, respectively.

### Extraction procedures

Two types of extraction systems were used (MAE and CSLE). The optimum conditions were determined as follows.

#### The effect of the choice of extraction solvent on recovery

Six different extraction solvent systems (n-hexane, acetonitrile, n-hexane-water, n-hexane-acetonitrile, n-hexane-acetone and dichloromethane-acetonitrile) were applied to find the combination with the highest extraction efficiency. BaP standard of 125 µg L^− 1^ and 10 mL of different solvent systems were added to the tea samples set for extraction. The extraction was performed twice in the microwave oven for 2 min.

#### Effect of different ratios of n-hexane-acetonitrile mixture on recovery

125 µg L^− 1^ BaP was added to tea samples that were then extracted twice for 2 min in the microwave and twice for 4 min in the CSLE using mixtures containing different amounts of n-hexane-acetonitrile solvents. The average % recovery of the BaP compound was then examined.

#### The effect of the number of extractions on recovery

MAE was applied for once and twice using different ratios of 10 mL n-hexane: acetonitrile (1:3, v/v) mixtures. CSLE was applied for once and twice using different ratios of 10 mL n-hexane: acetonitrile (3:1, v/v) mixtures.

#### The effect of extraction time on recovery

Microwave extraction was examined for four different times (1, 2, 3 and 4 min) using 10 mL n-hexane: acetonitrile (1:3, v/v) mixtures. Using 10 mL of n-hexane: acetonitrile (3:1, v/v) mixtures with classic extraction applied twice, the effect of classical extraction on the recovery for 1, 2, 3, 4 and 5 min extraction times were investigated.

### Finding % recovery values at different BaP concentrations added to tea samples

BaP standards of 10 and 1 µg L^− 1^ were added to tea samples. The samples were extracted twice within 2 min in a household microwave oven that was brought to 180 W beam emission using 10 mL of n-hexane-acetonitrile (1:3, v/v) mixtures.

### Statistical analysis

The two methods studied for BaP average concentrations were compared according to the t-test, a significant difference (*p* > 0.05) was found. All statistical calculations such as standard deviation, relative standard deviation and statistical tests were done using Excel program.

## Results and discussion

To develop a method that can determine BaP from black tea samples by HPLC-FLD, the parameters such as solvent composition, extraction time and number of extractions employed in both procedures were optimized. Tea samples were extracted via MAE and CSLE methods. The best BaP recovery percentages (in the range of 83–97% as a result of the extractions repeated twice for 2 min) were achieved using microwave-assisted extraction method with n-hexane: acetonitrile (1:3, v/v) as the solvent. The limit of detection (LOD) and limit of quantification (LOQ) values ​​were found as 0.16 and 0.55 µg kg^− 1^, respectively. The LOD meets the criteria as described in the European Union Commission Regulation (EU) no. 836/2011 (LOD < 0.30 and LOQ < 0.90 µg kg^− 1^) (Thi et al. 2020). The LOD and LOQ values found in our study are below the EU Commission criteria. The BaP concentration in black tea samples was found to vary in the range of 0.75–0.83 µg kg^− 1^ by microwave assisted extraction method.

Using the CSLE method, the best BaP recovery percentages (in the range of 81–104%) were attained using n-hexane: acetonitrile (3:1, v/v) solvent and extractions were repeated twice for 4 min. From the calibration graph drawn, the limit of detection (LOD) and limit of quantification (LOQ) values ​​were determined to be 0.16 and 0.55 µg kg ^− 1^ respectively, and the concentration of BaP varied in the range of 0.72–0.86 µg kg^− 1^.

The EU Commission (2011) has established regulated maximum levels of allowable PAHs on various food products, but none have been accepted and acknowledged for teas yet (Peng and Lim 2022) but according to the European Medicines Agency (EMA / HMPC / 300,551/2015), the maximum limit value of BaP is given as 10 µg kg^− 1^ for herbal preparations prepared by subjecting the whole plant or herbal parts to various processes such as extraction, fermentation or drying (HMPC 2016). In the present study, the BaP concentration range in tea content was seen to be well below the maximum acceptable limit.

### Optimization of extractions

#### Effect of selection of extraction solvent on recovery

A suitable extraction solvent can effectively extract the BaP. Extraction solvents with different polarities, namely: n-hexane: acetonitrile, n-hexane: acetone, n-hexane: dichloromethane, n-hexane: water, n-hexane and dichloromethane were investigated among which n-hexane: acetonitrile was found to yield the best recoveries. The average % recovery, standard and relative standard deviation values ​​of the BaP compound (where extraction was applied twice for 2 min) from tea samples prepared using different solvent systems and spiked with 125 µg L^− 1^ BaP standard are given in Table 1.

**Table 1:** The average % recovery and relative standard deviation values of BaP from tea samples in different solvent systems, 95% CL, (*N* = 9) (MAE applied was applied 2 times for 2 min)

Solvent systems	% recovery	Relative standard deviation
n-hexane-acetonitrile (3:1, v/v)	103	4
n-hexane-acetone (3:1; v/v)	123	1
n-hexane-water (3:1; v/v)	51	3
n-hexane	71	4
DCM	108	3
DCM: acetonitrile (3:1, v/v)	111	1

N: Number of measurements

BaP % recovery was calculated as 103 ± 4% in the extraction done using n-hexane: acetonitrile (3:1, v/v) mixture as the extraction solvent. This recoverability value was better than the values obtained from other solvent systems. Considering the high recoverability and low standard deviation, microwave extraction application was chosen for testing different ratios of the hexane: acetonitrile solvent mixtures. To compare the two methods, the selected solvent mixture was also applied to the classical method.

#### Effect of different ratios of n-hexane: acetonitrile mixture on recovery

Table 2 shows the average % recovery, relative standard deviation values ​​of BaP compound from tea samples prepared with mixtures containing different ratios of n-hexane: acetonitrile and the addition of 125 µg L^− 1^ BaP standard (MAE was applied twice for 2 min for each sample). n-hexane: acetonitrile (1:3; v/v) solvent system stood out among the different ratios of n-hexane: acetonitrile mixtures with a high recovery and low relative standard deviation (99 ± 4%). Hence it was chosen for optimization. It should be noted that n-hexane was not preferred even though it also produced good recoveries because when working alone in the laboratory it can be more toxic than acetonitrile (Joshi and Adhikari 2019).

**Table 2:** Average % recovery and relative standard deviation values of the BaP compound from tea samples in different solvent ratios, (*N* = 9)

Solvent ratios	% recovery (MAE, 2 times 2 min each)	Relative standard deviation	% recovery (CSLE, 2 times 4 min each)	Relative standard deviation
n-hexane-acetonitrile (1:3, v/v)	99	4	77	6
n-hexane-acetonitrile (3:1, v/v)	103	4	104	5
n-hexane-acetonitrile (1:1, v/v)	70	8	84	4

N: Number of measurements

Table 2 shows the mean % recovery and relative standard deviation values ​​of the BaP compound extracted by solvents prepared from different ratios of n-hexane: acetonitrile and samples spiked with 125 µg L^− 1^ BaP standard (CSLE was applied twice for 4 min for each sample). From the different ratios of n-hexane: acetonitrile mixtures, the system having the highest recovery and low relative standard deviation (104 ± 5%) for the n-hexane: acetonitrile (3:1, v/v) solvent system was chosen for optimization in the classic method.

#### The effect of the number of extractions on recovery

In Table 3, the mean % recovery and relative standard deviation values of the BaP compound are given for the tea samples spiked with BaP standard (MAE was applied once for 2 min).

**Table 3:** BaP mean % recovery, standard deviation and relative standard deviation values for tea samples extracted once, 95% CL, (*N* = 9)

Components of *n*-hexane-acetonitrile mixtures	% recovery (MAE, 2 min)	Relative standard deviation	% recovery (CSLE, 4 min)	Relative standard deviation
n-hexane-acetonitrile (1:3, v/v)	92	5	79	2
n-hexane-acetonitrile (3:1, v/v)	75	2	83	4
n-hexane-acetonitrile (1:1, v/v)	60	5	81	3

N: Number of measurements

It should be noted that the % recovery values of BaP decreased in the one-time MAE application but higher recovery values were obtained when the extraction application was repeated twice at 2 min intervals with n-hexane: acetonitrile solvent systems although more solvent was spent. Table 3 shows the mean % recovery and relative standard deviation values of BaP compound at 95% CL, (*N* = 9) (MAE applied) for tea samples extracted once for 2 min.

The average % recoverability values ​​of BaP from tea samples to which BaP standard was added, in which classical solid-liquid extraction process was applied once for 4 min are given in Table 3. The % recovery values of BaP decreased to the levels as low as 79%. in one-time extractions. For this reason, it was concluded that higher recovery values were only attainable with the n-hexane: acetonitrile (3:1, v/v) solvent system with two repeated extractions in 4 min. Hence, the extraction process may be recommended to be repeated twice for BaP determination.

#### The effect of extraction time on recovery

The average % recoverability, standard and relative standard deviation values of BaP from the tea samples added to the BaP standard, where different extractions were applied are given in Table 4. The highest retractability in MAE was achieved in 2 min (97 ± 2%). It is thought that the recoveries obtained during these periods reduced owing to the fact that splashes due to heating were observed in the flask at 3 and 4 min.

**Table 4:** Average % recoverability values of BaP in tea samples extracted by MAE and CSLE method twice at different times, 95% CL, (*N* = 9)

Exctraction time (min)	% recovery (MAE)	Relative standard deviation	% recovery (CSLE)	Relative standard deviation
1	28	1	85	10
2	97	2	87	8
3	28	1	117	4
4	23	2	104	5

N: Number of measurements

Table 4 shows the average % recovery values of BaP from tea samples spiked with BaP standard and where CSLE was applied twice at different periods. In CSLE, 4 min was chosen as the optimum time due to high recoverability and low relative standard deviation (104 ± 5%).

### Average % recovery values of tea samples with varying concentrations of added BaP standard

**Fig. 1:**
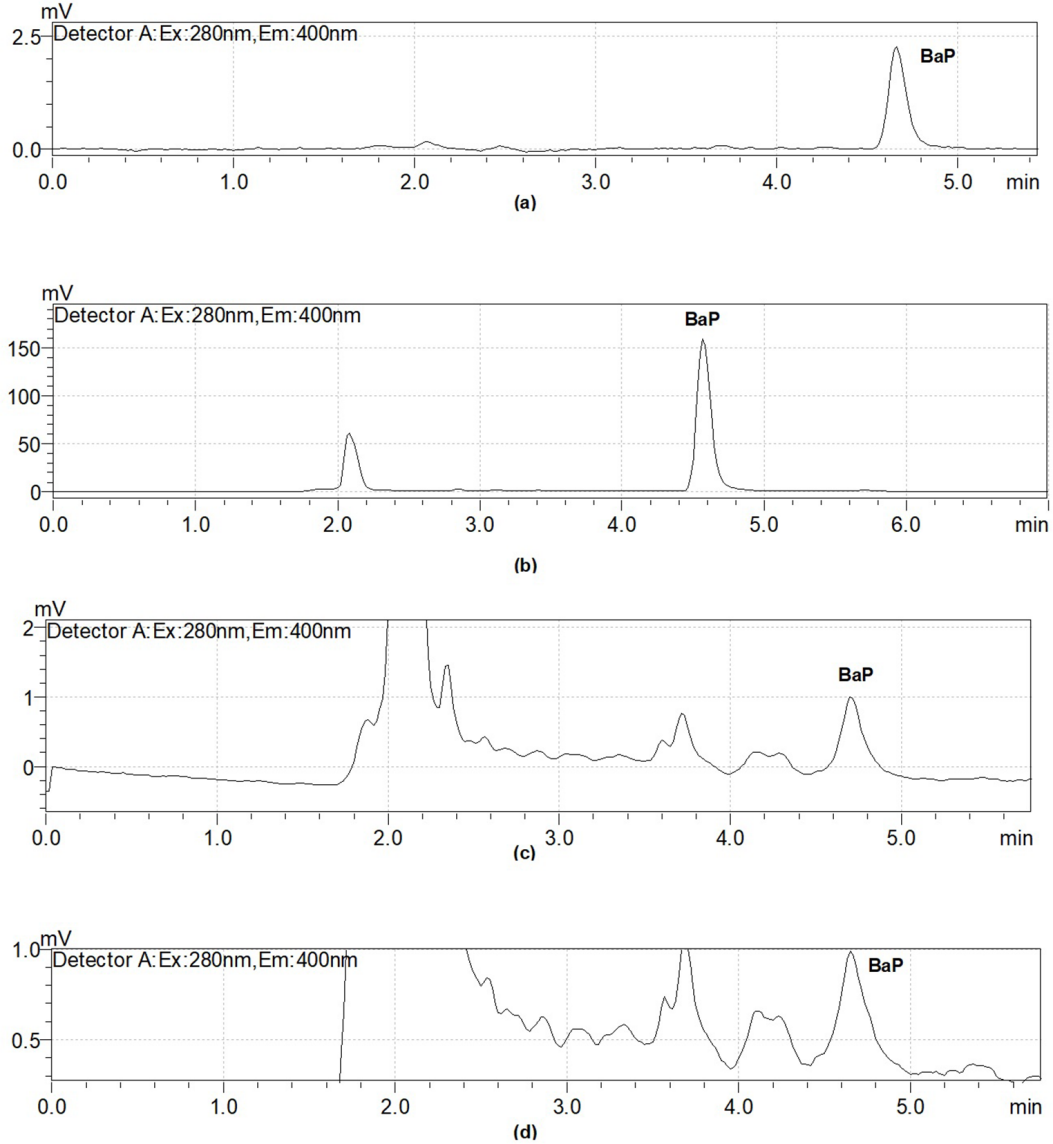
(**a**) HPLC chromatogram for BaP calibration standard (1 µg L^-1^). (**b**) HPLC chromatogram of the sample spiked with 1 µg L^-1^ BaP standard. (**c**) HPLC chromatogram of the tea sample (extracted under MAE conditions). (**d**) Tea sample HPLC chromatogram (solid-liquid extraction)

Average % recovery values of BaP compound obtained from both methods calculated from tea samples at different added BaP standard concentrations are shown in Table 5. The HPLC chromatogram of BaP standard at 1 µg L^− 1^ concentration is given in Fig. 1a. In Fig. 1b, HPLC chromatogram of the sample spiked with 1 µg L^− 1^ BaP standard is shown. The elution time of BaP (4.7 min) was compared with that of a standard BaP solution.

**Table 5:** Average % recovery of BaP compound for tea samples with different concentrations of BaP standard added by MAE and CSLE method. 95% CL, (*N* = 9)

Concentration standard added to tea sample (µg L^-1^)	% recovery (MAE)	Relative standard deviation	% recovery (CSLE)	Relative standard deviation
1	92	6	86	8
10	83	8	81	10
125	97	2	104	5

N: Number of measurements

Investigations were done for Microwave-assisted extractions performed twice for 2 min with the n-hexane: acetonitrile (1:3, v/v) solvent system on tea samples in which three different concentrations (1, 10 and 125 µg L^− 1^) were added. It was seen that the recoverability values ranged from 83 to 97%.

Tea samples with three different concentrations (1, 10 and 125 µg L^− 1^) of BaP added to them prepared for 4 min with n-hexane: acetonitrile (3:1, v/v) solvent system were also investigated. It was seen that the % recoverability values found as a result of classical solid-liquid extractions performed twice in a period of time varied between 81 and 104%.

### Investigation of the presence of BaP in real tea sample

In Fig. 1c, an HPLC chromatogram of a tea sample without BaP standard is given. BaP concentration in black tea samples was found to vary in the range of 0.75–0.83 µg kg^− 1^ (mean 0,792 ± 0,057 µg kg^− 1^) with microwave assisted extraction under optimized conditions. The mean levels of BaP in black tea samples grown and produced in the Eastern Black Sea region was lower than the reported value of 9.40 µg kg^− 1^ by Li et al. (2011), in China. Adisa et al. (2015) and Zachara et al. (2017) reported higher values than in our study ranging between 2 and 29 µg kg^− 1^ and 3.9–209 µg kg^− 1^ for BaP in black tea samples from their studies conducted in United States and Poland.

The range for the BaP concentrations in the brew obtained from 4 g of dry tea was found to be low (0.09–0.13 µg L^− 1^). It was thought that this might have been due to the low solubility of the BaP in water (hence the low rate of transition to tea brew). The BaP concentrations were found to be higher in our study in comparison to a study conducted in Spain where a value of 0,0036 µg L^− 1^ was reported by Mañana-López et al. (2021). On the other hand, Thi et al. (2020) reported BaP concentrations 0.58 µg kg^− 1^ in tea infusion in a study carried out in Vietnam where the values were lower than the values obtained in our study while Iwegbue et al. (2016) reported BaP concentrations ranging from 12.3 to 59 µg kg^− 1^ in tea infusions in a study carried out in Nigeria which were higher than values obtained in our study.

Figure 1d shows an HPLC chromatogram of the tea sample without added BaP standard where a classical solid-liquid extraction extraction was applied. The BaP concentration in black tea samples was found to change in the range of 0.72–0.86 µg kg^− 1^ (mean 0.797 ± 0.095 µg kg^− 1^) by CSLE at optimized conditions.

According to the t-test, there is no significant difference between the average concentration of BaP (t < t_crit_; 0.14 < 2.16 for BaP) obtained from classical extraction and microwave extraction.

## Conclusion

In order to develop a method that could be used to determine BaP from black tea samples by HPLC with fluorescence detection, the parameters in the extraction stage were optimized. Tea samples were extracted by MAE and CSLE methods. Parameters such as solvent composition, extraction time and number of extractions used in both extraction methods were optimized.

Although the composition of the tea is often complex, with the application of optimized extraction methods, the extraction process was carried out simply, cheaply, reliably and quickly without the need for a pre-cleaning step. BaP was detected in black tea by HPLC analysis of black tea samples with acetonitrile as the mobile phase. The MAE method was considered to be more advantageous in terms of time and solvent toxicity. Most of the solvents used for the extraction recycled in the rotary evaporation step. The MAE method was considered to be more advantageous in terms of time and solvent toxicity. Acetonitrile is less toxic solvent than hexane, the high ratio of acetonitrile in the solvent composition used in MAE makes this MAE method more attractive in a perspective from the human health. It has been observed that the optimum periods are 4 min in total in MAE and 8 min in classical extraction. The MAE method was considered to be more advantageous considering the time.

## Data Availability

All data presented in the manuscript is original and has not been published elsewhere.
